# The role of smoking as a risk indicator for apical periodontitis and endodontic status: a cross-sectional study of a portuguese adult sample

**DOI:** 10.1007/s10266-026-01330-6

**Published:** 2026-02-26

**Authors:** Isabel Silva Martins, Natália Pestana de Vasconcelos, Américo Santos Afonso, Ana Cristina Braga, Irene Pina-Vaz

**Affiliations:** 1 RISE-Health, Faculty of Dental Medicine, University of Porto, 4200-319 Porto, Portugal; 2 Health Science Faculty, University Fernando Pessoa, 4200-150 Porto, Portugal; 3 Faculty of Dental Medicine, University of Porto, 4200-319 Porto, Portugal; 4 ALGORITMI Research Centre, LASI, University of Minho, 4710-057 Braga, Portugal

**Keywords:** Apical periodontitis, Root canal treatment, Smoking, Tobacco

## Abstract

This study aimed to determine whether smoking acts as an independent predictor of apical periodontitis and endodontic treatment in a Portuguese adult population. This retrospective cross-sectional study was conducted in accordance with the PROBE (Preferred Reporting items for OBservational studies in Endodontics) guidelines. A total of 1841 first-time patient records were analyzed and met the inclusion criteria. Smoking status and systemic conditions were obtained from self-reported medical histories, and periapical status was assessed on panoramic radiographs using the Periapical Index (PAI). All assessments were conducted by calibrated observers who were blinded to clinical information. Data were analysed using IBM SPSS Statistics (Version 30.0; IBM Corp.) with descriptive, bivariate and multivariate logistic regression models, reporting odds ratios (OR) with 95% confidence intervals (CI) and significance at *p* < 0.05. Smoking was significantly associated with apical periodontitis (OR = 2.46; 95% CI: 1.96–3.11; *p* < 0.001), but not with endodontic treatment. Other factors independently associated with apical periodontitis included older age, greater caries experience, fewer remaining teeth, hypertension, and the presence of at least one root-filled tooth (OR = 3.19; 95% CI: 2.49–4.09; *p* < 0.001). Apical periodontitis showed the strongest association with the presence of at least one root-filled tooth (OR = 3.13; 95% CI: 2.47–3.96; *p* < 0.001). In conclusion, smoking may be considered a risk indicator for apical periodontitis. The increased inflammatory burden associated with periapical lesions highlights the importance of oral-health-related factors and behavioral habits in determining periapical status.

**Supplementary Information:**

The online version contains supplementary material available at https://doi.org/10.1007/s10266-026-01330-6.

## Introduction

Tobacco use remains widespread worldwide, making it a continuing public-health priority. According to the Global Burden of Disease (GBD) Study 2023, an estimated 1.10 billion people aged 15 and older were current smokers [1]. In 2023, smoking accounted for 5.81 million deaths and 161.3 million DALYs globally [1]. It ranked as the second leading risk factor for death in men (about 15.3%) and the eighth in women (about 3.1%). In Portugal, smoking was the second leading risk factor for premature death and disability in 2015 [2].

Smoking is closely associated with hypertension and significantly elevates the risk of death from renal failure, intestinal ischemia, hypertensive heart disease, and various respiratory and gastrointestinal disorders. Overall, smokers face a two- to threefold higher all-cause mortality compared with never-smokers [3]. Tobacco use also induces vasoconstriction and endothelial dysfunction, compromising microvascular circulation and reducing oxygen and nutrient delivery [4, 5]. These vascular changes, combined with altered fibroblast activity and reduced collagen synthesis [6], impair tissue repair and can lead to pulp necrosis [7]. Moreover, smoking disrupts immune function by impairing neutrophil activity, decreasing phagocytosis, and increasing oxidative stress and inflammation [8, 9].

Apical periodontitis (AP) is a localized inflammatory condition of the periradicular tissues, primarily caused by microbial infection of the root canal system [10]. It develops through a dynamic interplay between pathogenic microorganisms and the host immune response. When microbial stimuli persist, the chronic immune reaction leads to progressive periapical tissue damage and bone resorption as the host attempts to contain the infection [11]. Chronic AP is typically associated with a predominantly Gram-negative anaerobic microbiota, as well as elevated local levels of pro-inflammatory cytokines and mediators [12, 13].

Animal studies offer biologically plausible evidence that tobacco affects periapical status. Cigarette smoke inhalation has been shown to exacerbate AP by intensifying the inflammatory response and increasing pro-inflammatory cytokines, leading to greater tissue damage [14]. Moreover, experimental data suggest that AP may be aggravated not only by active smoking but also by chronic alcohol intake and secondhand smoke exposure, which have been linked to reduced hepatic antioxidant capacity and tissue alterations [15].

Despite this plausibility and the presence of shared risk factors between smoking and AP, human studies remain inconclusive. Some cross-sectional [16] and clinical prospective investigations [17] found no association between smoking and periapical status (i.e., prevalence of AP or endodontic treatment). Kirkevang et al. [18] reported that smoking was a statistically significant risk factor for AP, but its effect diminished after adjusting for age and marginal bone loss. Other authors have reported stronger associations between smoking and the prevalence of AP [19–21].

Findings from Portugal reflect the same uncertainty. Correia-Sousa et al. [22] reported a higher prevalence of AP and root-filled teeth (RFT) among smokers, in bivariate analyses, suggesting a potential association. However, Melo et al. [23] observed high rates of AP and RFT prevalence, regardless of systemic conditions or smoking habits. This inconsistency is further highlighted by a recent systematic review and meta-analysis of observational studies [24], which concluded that smokers have an increased prevalence of AP and root canal treatments, although the certainty of evidence was rated as moderate.

This is the first investigation in Portugal to comprehensively examine and update the relationship between periapical and endodontic status and smoking habits. To our knowledge, it is also the first Portuguese investigation to assess smoking as a risk indicator for apical periodontitis using multivariate analytical models. Moreover, this study expands upon previous national research by Correia-Sousa et al. [22] and Melo et al. [23] incorporating a substantially larger sample size, thereby providing more robust and generalizable findings. Accordingly, the primary objective of this cross-sectional study was to determine whether smoking is independently associated with AP in a Portuguese adult population. A secondary objective was to evaluate the potential influence of smoking on endodontic status, specifically the prevalence of RFT, compared with non-smokers. Both objectives were assessed after adjusting for relevant dental and systemic variables. The null hypothesis was that smoking is not independently associated with either AP or RFT.

## Materials and methods

### Study design and reporting guidelines

This was a retrospective, cross-sectional study and was conducted in accordance with the PROBE reporting guidelines [25]. The PROBE reporting checklist was included in supplement material (Table S1).

### Setting

The study was conducted at the Faculty of Dentistry of the University of Porto. Data were obtained from medical and dental electronic records of all patients attending their first visit to the pedagogic clinic between January and December 2018 (recruitment period). The exposure period corresponds to the same interval, as all variables were recorded at the initial visit. There was no follow-up because this was a retrospective cross-sectional study. Data extraction and collection were performed during the following year, using anonymized records for research purposes.

### Sample size

This study utilized a full cohort approach, encompassing all available electronic records of patients attending their first visit between January and December 2018. This approach is consistent with previous investigations in endodontic epidemiology, where either full cohorts or large convenience samples were used (e.g., Kirkevang et al. [18]: *n* = 1199; Sopińska, Bołtacz-Rzepkowska [20]: *n* = 815; Peršić Bukmir et al. [21]: *n* = 522). Including all eligible records (*n* = 2063) ensured maximum statistical power and avoided selection bias. No formal sample size calculation was performed because all eligible records from a defined period were included (full cohort approach), maximizing statistical power and minimizing selection bias.

### Patient selection and eligibility criteria

**Fig. 1:**
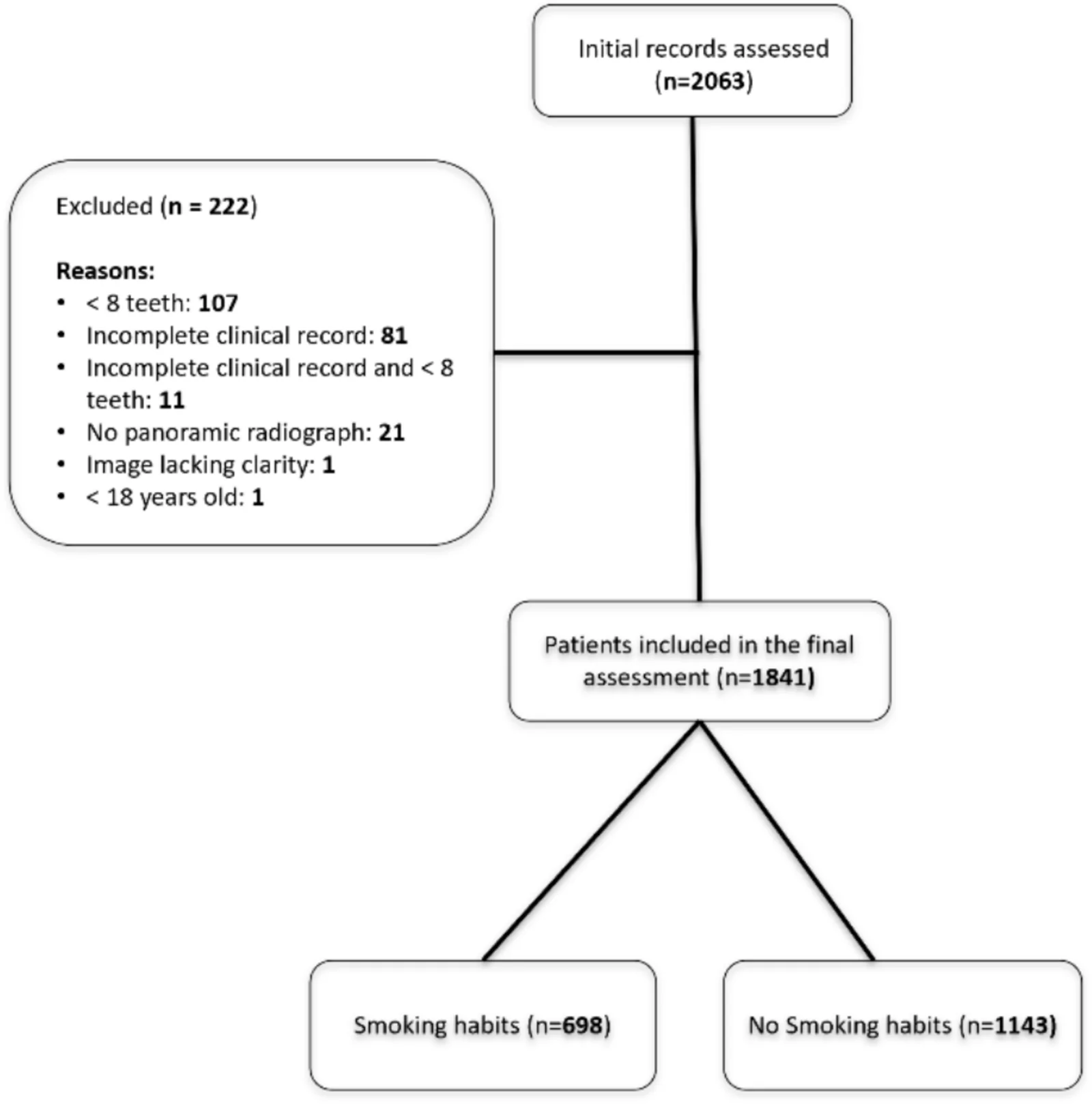
Flowchart illustrating participant inclusion and exclusion (Flowchart created using Microsoft PowerPoint)

Patient records were consecutively selected from individuals who sought routine dental care during the specified period. The inclusion criteria required patients to be aged 18 years or older, have more than eight teeth, present complete clinical records, and have a digital panoramic radiograph of sufficient diagnostic quality. Records that did not meet these criteria were excluded from the study. After applying the eligibility criteria, 1841 patient records met the inclusion criteria and were included in the final analysis, while 222 were excluded (Fig. 1).

### Ethical considerations

All patients signed written informed consent at their first visit to the Faculty of Dentistry, which included authorization for the use of anonymized clinical and radiographic data for research purposes. The consent forms are obtained and stored by the administrative services of the Faculty in secure institutional archives, with restricted access and in compliance with data protection regulations. The study protocol was approved by the Ethics Committee for Health of the Faculty of Dentistry (Research Project No. 13/2025).

### Data collection and assessment methods

A comprehensive dataset, including sociodemographic variables (age and gender), behavioral habits (smoking), and health-related conditions, was obtained from patients’ clinical charts. Hypertension, diabetes mellitus (DM), and smoking habits were recorded from self-reported medical histories using dichotomous (yes/no) responses. For smoking status, patients were classified as non-smokers if they had never smoked, and as smokers if they were current or former smokers, following previous studies [26, 27]. Smoking status was self-reported, which may introduce exposure misclassification. Additionally, smoking intensity and duration were not recorded, representing potential residual confounding. Regarding hypertension, stricter criteria were applied; only patients taking antihypertensive medication were considered hypertensive [27]. Data extraction was carried out manually by trained investigators during the year following the study period.

Radiographic data collection was performed using the digital panoramic radiographs available in the clinical records. Two experienced endodontists (NV and IM), each with over 20 years of clinical practice, independently assessed the images using a medical-grade monitor in a standardized, dim-light environment. When needed, disagreements were resolved through consensus with a third evaluator, a senior professor of Endodontics (IPV). All observers were blinded to patients’ medical histories. All teeth were evaluated except third molars and dental implants.

Efforts to reduce bias included blinded radiographic assessment, observer calibration, standardized extraction protocol, and strict diagnostic criteria.

### Variables and outcomes assessed

*Primary outcome*: Apical Periodontitis (AP)—defined according to the Periapical Index (PAI) system [28]. Scores ≥ 3 were considered indicative of AP.

*Secondary outcome*: Root-filled teeth (RFT)—Teeth exhibiting radiopaque filling material in at least one root canal.

### Additional variables/exposures/predictors

Primary exposure: smoking status (smoker vs. non-smoker), defined as described previously. Additional predictors and potential confounders: age (continuous), gender, systemic conditions (hypertension, DM), and dental variables (teeth present, caries experience, residual roots).

The prevalence of AP, RFT, root-filled teeth with AP (RFT-AP), and untreated teeth with AP (UT-AP) was defined as the percentage of individuals presenting at least one affected tooth. All definitions followed methodologies used in previous epidemiological studies [23, 29, 30].

### Periapical status assessment and radiographic evaluations

The condition of the periapical tissues was assessed through orthopantomographs using the Periapical Index (PAI) scoring system proposed by Orstavik et al. [28]. For multi-rooted teeth, the highest root score was recorded as the overall PAI score for the tooth. All panoramic radiographs were obtained using an Orthopantomograph 9200 DDE (Gendex) and evaluated with VixWin Platinum software, which allows image magnification and contrast adjustment to facilitate interpretation.

### Observers’ calibration

Two observers with extensive endodontic experience (NV and IM) underwent calibration for the PAI system before evaluating the radiographs. The calibration involved scoring 100 radiographic images—both root-filled and non-root-filled—using the visual references for the five PAI categories [28]. Their scores were compared with a gold-standard atlas, yielding Cohen’s kappa values of 0.79 and 0.85, respectively.

Intraobserver reproducibility was then assessed: each examiner scored the panoramic radiographs of 50 randomly selected patients and repeated the scoring 2 months later after recalibration. The resulting intraobserver kappa values were 0.72 and 0.70. Interobserver agreement for PAI scoring produced a Cohen’s kappa of 0.71. In cases of disagreement, a final score was reached by consensus with a senior Endodontics professor.

### Statistical analysis

Data were initially recorded in Microsoft Excel (Microsoft Corporation) and then imported into IBM SPSS Statistics (Version 30.0; IBM Corp.). Descriptive statistics were presented as mean ± standard deviation (SD) for continuous variables and as frequencies and percentages for categorical variables. Bivariate analyses comparing smokers and non-smokers were performed using the Student’s *t*-test for normally distributed continuous variables and Pearson’s Chi-square test for categorical variables, with a 95% confidence interval (CI) and significance level *α* = 0.05. To evaluate whether smoking acted as a risk indicator of AP and RFTs, multivariate logistic regression analyses were performed. Outcomes were coded as 0 = absent and 1 = present. The primary exposure was smoking status (0 = non-smoker, 1 = smoker). Potential confounders (age, gender, systemic conditions, and dental variables) were selected based on biological plausibility. Multivariate logistic regression was performed using Backward Stepwise Elimination (BWSE). For this analysis, the threshold for remaining in the model was set at *p* < 0.05 for removal. Odds ratios (ORs) with 95% CIs were calculated, and *p*-values < 0.05 were considered statistically significant.

Missing data were addressed using a complete-case analysis. Records with incomplete clinical or radiographic information were excluded during eligibility screening (Fig. 1). No imputation was performed. No subgroup or interaction analyses were prespecified or conducted, as the study objectives focused on overall associations in the full sample. Category boundaries were defined as follows: AP was classified as PAI ≥ 3; smoking status as smoker (current or former) versus non-smoker (never smoked); hypertension as patients on antihypertensive medication; and DM as self-reported.

## Results

Data from 1841 patient records were analysed. A summary of the descriptive demographic and dental characteristics at the individual level is presented in Table 1. Participants’ ages ranged from 18 to 88 years. The prevalence of AP was 47.1%, indicating that nearly half of the participants had at least one affected tooth. Regarding endodontic status, 48.3% of the individuals had at least one root-filled tooth (prevalence of RFT). Among these, 26.8% had at least one root-filled tooth (RFT) with AP (RFT-AP). Additionally, 30.5% of participants presented AP in untreated teeth (UT-AP).

**Table 1:** Descriptive characteristics of the study participants

Variables	Total
Age (years), mean ± sd	43.83 ± 17.55
Gender, *n* (%)	
Male	761 (41.3)
Female	1080 (58.7)
No. of teeth with AP, mean ± sd	0.94 ± 1.31
Prevalence of AP, *n* (%)	867 (47.1)
No. of RFT, mean ± sd	1.17 ± 1.79
Prevalence of RFT, *n* (%)	889 (48.3)
Prevalence of RFT-AP, *n* (%)	494 (26.8)
Prevalence of UT-AP, *n* (%)	561 (30.5)
No. of teeth, mean ± sd	23.79 ± 5.12
Caries experience, mean ± sd	5.39 ± 4.57
No. of residual roots, mean ± sd	0.42 ± 2.03
Hypertension, *n* (%)	377 (20.5)
Diabetes mellitus, *n* (%)	123 (6.7)
Smoking Habits, *n* (%)	698 (37.9)

*sd* Standard deviation, *AP* Apical periodontitis, *RFT* Root-filled teeth, *RFT-AP* Root-filled teeth with apical periodontitis, *UT-AP* Untreated teeth with apical periodontitis.

Table 2 presents the distribution of the examined variables between the non-smoking group (never smokers) and the smoking group (ever or current smokers). The prevalence of AP and RFT was 64.3% and 55.7%, respectively, in the smoking group, compared with 36.6% and 43.7% in the non-smoking group (*p* < 0.001). In contrast, the prevalence of AP in RFT (RFT-AP), was higher among non-smokers (79.3%) than smokers (63.2%; *p* < 0.001). Similarly, the prevalence of AP in untreated teeth (UT-AP) was higher among non-smokers (78.4%) than smokers (55%; *p* < 0.001).

**Table 2:** Distribution of demographic, dental, and health-related variables by smoking status (bivariate analysis)

Variable	No. smoking habits	Smoking habits	*p*-value*
Age (years), mean ± sd	42.1 ± 18.2	46.6 ± 16.0	<0.001
Gender, *n* (%)			
Male	382 (33.4)	379 (54.3)	<0.001
Female	761 (66.6)	319 (45.7)	
No. of teeth with AP, mean ± sd	0.6 ± 1.0	1.4 ± 1.6	<0.001
Prevalence of AP, *n* (%)	418 (36.6)	449 (64.3)	<0.001
No. of RFTs, mean ± sd	1.0 ± 1.8	1.4 ± 1.8	<0.001
Prevalence of RFTs, *n* (%)	500 (43.7)	389 (55.7)	<0.001
Prevalence of RFT-AP, *n* (%)	906 (79.3)	441 (63.2)	<0.001
Prevalence of UT-AP, *n* (%)	896 (78.4)	384 (55.0)	<0.001
No. of teeth, mean ± sd	24.7 ± 4.6	22.4 ± 5.6	<0.001
Caries experience, mean ± sd	5.1 ± 4.6	5.9 ± 4.5	<0.001
Number of residual roots, mean ± sd	0.4 ± 2.2	0.5 ± 1.6	0.046
Hypertension, *n* (%)	188 (16.4)	189 (27.1)	<0.001
Diabetes mellitus, *n* (%)	60 (5.2)	63 (9.0)	0.002

*AP* Apical periodontitis, *RFT* Root-filled teeth, *RFT-AP* Root-filled teeth with apical periodontitis, *UT-AP* Untreated teeth with AP.

*Statistical significance: *p* < 0.05.

To examine variables related to oral status (number of teeth present, caries experience, and number of residual roots), a Student’s *t*-test was performed. Smokers had fewer teeth (*p* < 0.001) and greater caries experience (*p* < 0.001); however, there was no significant difference in the number of residual roots.

Regarding systemic health, both hypertension (27.1% vs. 16.4%; *p* < 0.001) and DM (9.0% vs. 5.2%; *p* = 0.002) were more prevalent among smokers.

To examine risk indicators for AP, a multivariate logistic regression analysis was performed, including demographic, clinical, and behavioral variables. As shown in Table 3, older age, greater caries experience, fewer remaining teeth, smoking, hypertension, and the presence of at least one root-filled tooth were independently associated with higher odds of AP. Smoking and the presence of root-filled teeth demonstrated the strongest associations with AP.

**Table 3:** Multivariate logistic regression (backward stepwise selection) for prevalence of AP (at least one tooth affected per individual; present vs. absent)

	B	SE	*p*	OR	95% CI for OR	
					Lower	Upper
Age (years)	0.016	0.005	<0.001	1.016	1.007	1.026
Caries experience	0.101	0.015	<0.001	1.107	1.075	1.139
No. of residual roots	0.232	0.064	<0.001	1.261	1.112	1.430
No. of teeth	−0.077	0.015	<0.001	0.926	0.900	0.953
Smoking habits	0.902	0.118	<0.001	2.464	1.955	3.106
Hypertension	0.686	0.161	<0.001	1.986	1.448	2.725
RFT	1.161	0.127	<0.001	3.192	2.489	4.093
Constant	−0.718	0.503	0.154	0.488		

*B* Regression coefficient, *SE* Standard error, *p* *p*-value, *OR* Adjusted odds ratio, *CI* Confidence intervals

*Statistically significant (*p* < 0.05).

To examine factors associated with the presence of root-filled teeth (RFT; at least one affected tooth per individual), a multivariate logistic regression analysis was performed. As shown in Table 4, age, gender, caries experience, and the presence of AP were significantly associated with the likelihood of having at least one root-filled tooth. Among these variables, the presence of AP showed the strongest association with RFT (Table 4).

**Table 4:** Multivariate logistic regression (backward stepwise selection) for prevalence of root-filled teeth (RFT; at least one tooth affected per individual; present vs. absent)

	B	SE	*p*	OR	95% CI for OR	
					Lower	Upper
Age (years)	0.018	0.003	<0.001	1.018	1.012	1.025
Gender	−0.318	0.117	0.007	0.728	0.578	0.916
AP	1.141	0.120	<0.001	3.129	2.472	3.961
Caries experience	0.282	0.017	<0.001	1.326	1.283	1.371
Constant	−2.441	0.246	<0.001	0.087		

*B* Regression coefficient, *SE* Standard error, *p* *p*-value, *OR* Adjusted odds ratio, *CI* Confidence intervals

*Statistically significant (*p* < 0.05)

## Discussion

The main findings of this study are that smoking is independently associated with AP, whereas no independent association was observed between smoking and the prevalence of RFT. These results partially support the null hypothesis, which stated that smoking would not be independently associated with either AP or RFT.

This study investigated the prevalence of AP and RFT in first-time dental patients at the Faculty of Dentistry of the University of Porto (2018). The study population was diverse in terms of age, educational background, place of residence, and socioeconomic status, reflecting the broad demographic profile of individuals seeking care in this setting. The investigation further examined whether smoking habits are independently associated with AP or RFT. Specifically, it assessed the association between smoking status and the prevalence of AP and RFT after adjusting for key dental and systemic variables, including age, gender, caries experience, number of present teeth, number of residual roots, DM, hypertension, and endodontic status.

As in several previous studies based on self-reported data, the smokers’ group in the present investigation included both former and current smokers [20, 22, 23]. In contrast, other authors excluded former smokers entirely [19, 21, 27], while some [20] classified as smokers only those who were tobacco-dependent at the time of the study and who had smoked at least 10 cigarettes per day for a minimum of five consecutive years. Although the duration and intensity of exposure to tobacco smoke have been reported as potential factors influencing the prevalence of AP [31], it is important to note that even very low cigarette consumption increases the risk of coronary heart disease and stroke. Evidence consistently demonstrates that no level of smoking is safe for cardiovascular health [32].

The study revealed a significant association between smoking habits and multiple oral and systemic health variables (Table 2). As is widely documented in the literature, smokers show a higher prevalence of AP [18, 21], while other studies report a higher prevalence of both AP and RFT [19, 20, 22]. This is consistent with the pro-inflammatory effects of tobacco exposure and the greater burden of oral disease typically observed in this population. Smokers also tend to undergo more dental treatments and experience higher rates of tooth loss and endodontic pathology [33]. Accordingly, in the present sample, former and current smokers had a lower mean number of teeth present and a greater caries experience compared with non-smokers, reflecting a poorer overall oral status. While cross-sectional data cannot establish causality, the results contribute to the broader evidence on the link between smoking and periapical pathology.

In the bivariate analysis, smokers showed a higher overall prevalence of AP and RFTs. In contrast, non-smokers exhibited higher proportions of RFT-AP and UT-AP. At first glance, this finding may appear counterintuitive when compared with the results of the adjusted models. However, bivariate analyses do not account for potential confounding factors and may therefore obscure or distort underlying associations that only become evident after multivariate adjustment. Several alternative explanations may account for these observations. Differences in treatment-seeking behavior, access to dental care, or the timing of dental interventions may influence the distribution of treated versus untreated periapical lesions between smokers and non-smokers. In addition, variations in treatment quality, lesion severity, or chronicity could contribute to the higher proportion of RFT-AP or UT-AP observed among non-smokers in unadjusted analyses. Smokers, for example, may present with more advanced disease or delayed care, whereas non-smokers may be more likely to receive endodontic treatment, resulting in a different cross-sectional distribution of periapical findings.

To account for these factors, a multivariate logistic regression was performed with AP and RFT as the dependent variables. Hypertension, smoking habits, age, carious experience, the number of residual roots, the number of teeth present, and prevalence of RFT remained significantly associated with AP, although each factor contributed differently to the overall risk (Table 3). Among these variables, the prevalence of RFT showed the strongest association with AP, followed by smoking habits and hypertension. Smokers exhibited approximately a 2.5-fold higher likelihood of having AP compared with non-smokers. These findings are consistent with those of a previous investigation by Peršić Bukmir et al. [34], which reported that although dental variables—such as the number of RFT and the presence of primary caries—were the strongest predictors of periapical status, daily smoking remained a significant predictor of periapical disease in the final multiple regression model. Similarly, studies conducted in Portugal [22], Spain [19], Denmark [18], Poland [20], and other international settings have reported a significant association between smoking habits and AP, supporting smoking as an important correlate of AP [35]. Complementary evidence comes from studies in the adult Croatian population [21]. Although this study did not find a significant difference in the prevalence of AP between smokers and non-smokers at the individual level, the authors did observe a notable discrepancy when analysing the fraction of teeth affected by AP: on average, smokers had two more teeth with AP than non-smokers.

In the multivariate regression analysis, among the systemic conditions considered (hypertension and DM), only hypertension remained significant in the final model. Globally, approximately one-third of adults are affected by hypertension, yet fewer than half of those aware of their condition actively manage it [36]. In our sample, hypertensive patients showed nearly twofold higher odds of presenting with AP (OR = 1.98). This finding aligns with previous evidence noting that cigarette smoking increases the risk of developing hypertension [37]. Although our study did not specifically assess hypertensive smokers as a separate subgroup, the combined evidence from the literature suggests that individuals with hypertension—particularly if they also smoke—may be more susceptible to AP, consistent with observations reported by Segura-Egea et al. [27].

Other investigations have shown a clear association between DM and oral pathology, including fewer teeth and a higher number of untreated (UT-AP) or RFT with AP [38]. Differences in criteria—such as including only medicated or hospitalized diabetic patients—may influence the results. A recent meta-analysis [39] evaluating whether DM influences the occurrence and progression of AP found that patients with DM show a higher prevalence of AP after root canal treatment, and that DM negatively influences treatment outcomes. DM was identified as a risk factor for impaired periapical healing. However, the authors noted several limitations, including incomplete data in some studies, which hindered the adjustment for confounders such as gender, smoking status, and variations in healing criteria, potentially introducing bias.

Regarding the potential association between smoking habits and the prevalence of RFT, our general linear analysis revealed a higher frequency of RFT among smokers (Table 2), consistent with previous studies [19, 20, 22]. However, smoking was not retained as a significant variable in the multivariable regression model (Table 4). These findings align with Peršić Bukmir et al. [21], who reported no association between smoking and RFT and observed that, after adjusting for age and gender, a higher proportion of endodontically treated teeth was actually associated with less AP, suggesting that treatment quality may be more decisive for periapical status than smoking habits. Jansson [26], investigating the relationship between AP in RFT and marginal bone loss, also found a significant correlation between smoking and the prevalence of RFT with AP; nevertheless, their multivariable analysis indicated that greater marginal bone loss—rather than smoking—was the factor significantly associated with AP. Other authors have described a significant association between smoking and the prevalence of RFT with AP, although the overall scientific evidence points to a significant but weak relationship [40].

Panoramic radiographs were used to assess periapical status, as full-mouth intraoral surveys are rarely performed in routine clinical settings. Although panoramic imaging has lower precision than more recent imaging modalities such as CBCT [41], which also involves higher radiation exposure, it remains widely accepted in epidemiological studies [21]. Moreover, panoramic radiographs have demonstrated a sensitivity of 86–96% for detecting AP compared to full-mouth surveys [42]. Its clinical advantages include low radiation exposure, rapid acquisition, and the ability to visualize all teeth and supporting structures in a single image. Moreover, this method provides valuable diagnostic information regarding caries, restorations, RFT, and periapical conditions [43], making it a practical and informative tool for large-scale studies. To ensure greater accuracy in identifying teeth with AP, the observers were calibrated to use the Periapical Index (PAI) system [28]. Intra- and inter-observer Cohen’s kappa values were comparable to those reported in a previous study [34].

Patient selection included all first-time consultations from 2018 that met the eligibility criteria, making the sample representative of the clinic population for that year and consistent with previous Portuguese [22, 23, 44] and international studies [19, 27]. The final sample was large (*n* = 1841), exceeding many comparable investigations, and the mean number of teeth (23.8 ± 5.1) reflected moderate tooth loss. Although not fully representative of the broader Porto population, the substantial sample size improved the precision of periapical status estimates, particularly among smokers. Individuals with eight or fewer teeth were excluded to avoid confounding from advanced periodontal breakdown [22, 27, 45]. The use of standardized inclusion criteria supports the methodological robustness of the study, despite the known limitations of panoramic radiography in detecting early periapical lesions [20]. Additionally, the use of the PROBE guidelines in the design and reporting of this cross-sectional study enhances the reliability and reproducibility of the findings, providing a structured framework that strengthens the validity of observational research in Endodontics.

Cross-sectional studies have inherent limitations in controlling for confounding factors and establishing causality. Differences in patients’ socio-economic status, oral hygiene practices, access to dental care, systemic health conditions, and variables such as the presence of AP prior to root canal treatment or the quality of the treatment itself may influence the outcomes. These constraints warrant caution when generalizing the results to broader populations. Nevertheless, several strategies were employed to minimize potential sources of bias. Radiographic assessments were independently performed by two experienced endodontists, with both intra- and inter-observer calibration; a third evaluator was consulted to reach consensus when necessary. Observers were blinded to patients’ medical histories to prevent information bias. Strict diagnostic criteria were applied to clinical variables (e.g., hypertension was recorded only in individuals who were medicated), and regression models were adjusted for potential confounders. To address concerns regarding missing or incomplete data [9], the reasons for patient exclusions were explicitly documented, and a flowchart was provided to illustrate the study enrolment process. Overall, despite the inherent limitations of the cross-sectional design, the rigorous methodology, careful calibration of radiographic assessments, adjustment for potential confounders, and transparent handling of missing data support the internal validity of the study findings.

The generalizability of our findings should be interpreted with caution, as the results reflect the characteristics of the population studied and may not fully apply to other settings with different demographic or systemic health profiles. Nevertheless, the consistency of our observations with previous reports supports their potential external validity. Importantly, given the cross-sectional design, the observed associations should not be interpreted as evidence of causality.

Implications for future research are clear. Further longitudinal studies on the effect of smoking on periapical and endodontic status are needed, ideally with precise documentation of smoking habits and rigorous control of confounding factors related both to the individual and to the endodontic treatment itself. Such research is particularly important given the substantial social burden of these conditions, which are associated with significant morbidity and reduced quality of life.

Smoking, a highly prevalent and modifiable behavior, was independently associated with AP in this study. This observed association highlights the clinical relevance of smoking for periapical health and underscores the importance of early detection and management of caries and its complications in affected patients. The increased inflammatory burden associated with AP further emphasizes the need for proactive oral care. Clinicians should remain attentive to patients’ smoking habits and support cessation strategies, as smoking may be considered a risk indicator for AP. While hypertension and other systemic factors can also influence periapical outcomes, the results of this study reinforce smoking as a key behavioral factor that dental professionals can address in routine practice to help improve oral and overall health outcomes.

## Conclusions

Smoking may be considered a risk indicator for AP in Portuguese adults. The increased inflammatory burden associated with periapical lesions highlights the importance of oral health-related factors and behavioral habits. While smoking represents a modifiable behaviour requiring continued attention, clinicians should also consider systemic conditions such as hypertension, as well as age, gender, and overall oral status, all of which can influence periapical outcomes. Dental professionals should remain attentive to patients’ systemic profiles, support smoking-cessation efforts, and manage other systemic conditions, emphasizing the relationship of these factors with periapical health. Given the cross-sectional design, causality cannot be inferred, and longitudinal studies are needed to confirm these associations.

## Supplementary Information

Below is the link to the electronic supplementary material.

Supplementary file1 (Docx 54 KB)

## Data Availability

All data included in this current study are available from the corresponding author upon request.
